# Congenital Hypothyroidism: Space–Time Clustering of Thyroid Dysgenesis Indicates a Role for Environmental Factors in Disease Etiology

**DOI:** 10.1089/thy.2020.0005

**Published:** 2021-06-08

**Authors:** Richard J.Q. McNally, Jeremy H. Jones, Mohamad Guftar Shaikh, Malcolm D.C. Donaldson, Karen Blakey, Tim D. Cheetham

**Affiliations:** ^1^ Population Health Sciences Institute, Newcastle University, Sir James Spence Institute, Royal Victoria Infirmary, Newcastle upon Tyne, United Kingdom.; ^2^ Royal Hospital for Children, Glasgow, United Kingdom.; ^3^ Section of Child Health, Royal Hospital for Children, University of Glasgow School of Medicine, Glasgow, United Kingdom.; ^4^ Department of Paediatric Endocrinology, Royal Victoria Infirmary, Newcastle upon Tyne, United Kingdom.; ^5^ Translational and Clinical Research Institute, Newcastle University, Newcastle upon Tyne, United Kingdom.

**Keywords:** congenital hypothyroidism, environment, etiology, space–time clustering, thyroid dysgenesis

## Abstract

***Background:*** The etiology of most cases of congenital hypothyroidism (CHT) due to thyroid dysgenesis (DG) is unknown. If transient environmental factors can impact on thyroid gland development, then clustering of cases in time and/or space may occur, and this would be more likely in thyroid DG than dyshormonogenesis (DHG).

***Methods:*** The newborn screening program for CHT in Scotland is linked to a central database that includes case details such as postcode. The etiology of CHT is investigated in many cases of CHT using scintigraphy and/or ultrasonography. We looked for evidence of a change in CHT incidence with year of birth and according to season of the year. We then undertook space–time clustering analysis (using a method based on *K*-functions, with nearest neighbor thresholds) of CHT in Scotland between 1979 and 2015. We also looked for evidence of overall changes associated with sex and area-based birth density.

***Results:*** Of 531 cases with CHT during the study period, 290 cases had been categorized as DG (*n* = 229) or DHG (*n* = 61) following more detailed investigation. The incidence of CHT increased with year of birth and was in part linked to changing methodology, but there was no seasonality. There was no evidence of overall space–time clustering (*p* = 0.06), but there was evidence of clustering in babies with DG (*p* = 0.007). This picture appeared to be most closely linked to underlying thyroid gland hypoplasia rather than thyroid gland agenesis or ectopia. There was significant space–time clustering for both males and females, but clustering was restricted to lesser birth density areas. There was also evidence of clustering for unknown cases (*p* < 0.001). Clustering of these cases was restricted to females but was present for cases from both greater and lesser birth density areas. There was no evidence of clustering in cases of DHG.

***Conclusions:*** These data suggest that an unidentified environmental factor or factors may be involved in the etiology of thyroid DG in Scotland. The variation in CHT incidence observed internationally may reflect environmental as well as genetic factors.

## Introduction

Congenital hypothyroidism (CHT) is the most common neonatal endocrine disorder. Around 80% of CHT cases have been attributed to thyroid dysgenesis (DG) where the thyroid gland is absent, ectopic or hypoplastic. The remaining cases have been linked to dyshormonogenesis (DHG) where a normally situated thyroid gland harbors an enzyme defect. The etiology of most cases of DG is unknown with a small number of genes implicated (1,2). DHG is usually autosomal recessive, and analyses of genes in patients with DHG identify mutations in genes involved in thyroid hormone synthesis in a substantial proportion of cases (3).

Many CHT screening programs rely on identifying babies with elevated blood spot thyrotropin (TSH) concentrations. The TSH screening cutoff used has tended to fall with time and with an associated increase in CHT incidence from ∼1 in 4000 to 1 in 2000 (4). There is, nevertheless, evidence that the true incidence of CHT has risen over the past 40 years (5). Many of the additional cases of CHT detected lie at the mild end of the biochemical spectrum and have a gland *in situ* rather than DG (6). Some of these mild cases have an underlying DHG, and the proportion of all cases of CHT with an underlying DHG appears to be greater than previously thought. The etiology of most cases of unequivocal CHT due to DG remains unclear, as does the etiology in many cases at the mild end of the biochemical spectrum with a gland *in situ* (3). Many babies with a gland *in situ* have transient CHT, but this does not preclude an underlying molecular defect (7).

Space–time clustering occurs when excess disease cases are seen in small spatial locations at limited points in time. Space–time clustering cannot be attributed to underlying spatial fluctuations in disease incidence or change in incidence with time. We have described space–time clustering of babies born with an elevated TSH in northern England (8), suggesting that environmental factors are involved in CHT. One of the study limitations was that babies were only identified as having raised TSH concentrations. We therefore examined how CHT cases occur in relation to space and time in another part of the United Kingdom where patient numbers were larger, case ascertainment was high, and where a subgroup of babies were well characterized into likely DG or DHG. Our hypothesis was that the distribution of babies with DG would demonstrate space–time clustering.

## Materials and Methods

**FIG. 1.**
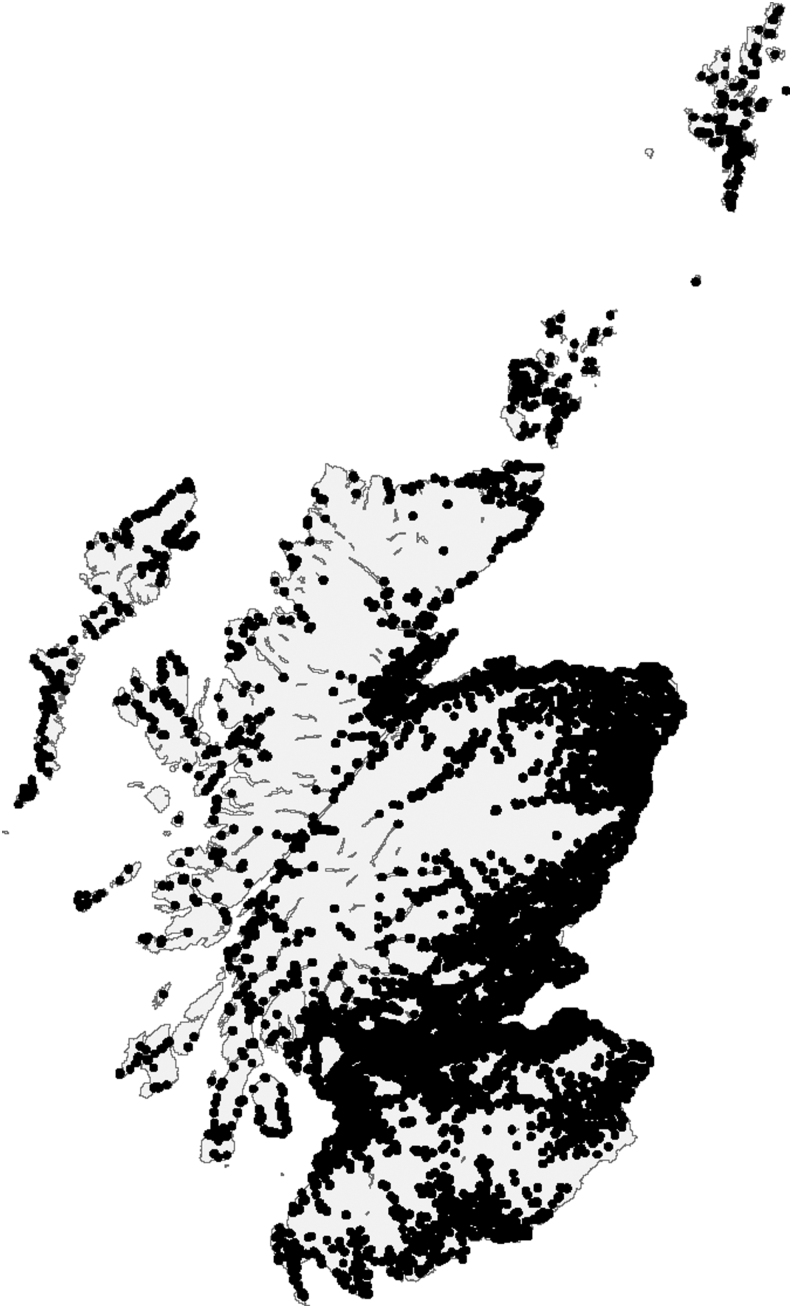
Map displaying the distribution of population centroids throughout the whole of Scotland (i.e., geographical centers of population).

A record has been kept of babies in Scotland referred with TSH elevation on neonatal screening since August 1979 (9). Glasgow is the screening center for Scotland, receiving blood spot samples taken on days 4–7. Figure 1 shows the population distribution in Scotland where babies are born. Ascertainment is greater than 95% with one of the study team (J.H.J.) compiling the database. Babies with CHT in the vicinity of the largest conurbation (Glasgow) undergo investigation with ultrasonography and isotope scanning to establish whether they have DG or DHG. Referrals to Glasgow for imaging are made from some areas, while others use scintigraphic investigation, which is sometimes supplemented by ultrasonography.

### Cases that screen positive

The positive blood spot notification process has been described previously (10). Babies with a TSH value ≥25 mU/L in whole blood screen positive and undergo further testing. This includes thyroid function testing on serum and, in babies referred to the Glasgow unit, thyroid ultrasonography and radioisotope imaging with diagnostic imaging were also used in cases referred to three other major referral centers.

From 1979 until 1982, a radioimmunoassay (RIA) from Corning Diagnostics Ltd. (Sudbury, United Kingdom) was employed to determine blood spot TSH concentration. This was replaced by an in-house RIA (1982–1989), which was superseded by an immunoradiometric assay manufactured by IDS (Boldon, United Kingdom) from 1989 until 2002. Respective negative, repeat testing limits, and immediate referral concentrations of TSH for these assays were: <25, 25–49, ≥50 mU/L; <15, 15–39, ≥40 mU/L; and <10, 10–39, ≥40 mU/L (10). Since 2002, when the AutoDELFIA blood spot TSH method was adopted in Scotland, repeat testing was performed for babies with a blood spot TSH value between 8 and 25 mU/L. If repeat values are >8 mU/L, then babies are referred for formal blood tests and imaging in most centers (10). When methodology changed, revised cutoffs with the updated assay were established following linear regression analysis against the old assay.

### Thyroid imaging

Ultrasonography and isotope scanning characterize babies into DG or babies more likely to have DHG (11,12). DG is characterized by absent, ectopically situated, or a small thyroid gland *in situ*, while DHG is diagnosed on the basis of a normal or enlarged *in situ* gland on ultrasonography and/or increased uptake on isotope scan (13). DHG was inferred when two or more siblings were affected with CHT in the context of a normal-sized or large eutopic gland. Thyroid volumes on ultrasound were compared with normative data (14) and deemed to be of normal size or greater (in keeping with DHG) or hypoplastic in keeping with DG. Sonographers did not access isotope reports at the time of scanning. Isotope scanning was carried out after an intravenous injection of 10–14 MBq 99m-technetium pertechnetate.

### Diagnostic categories

The diagnosis of CHT was made on the basis of biochemistry using previously published diagnostic criteria (9). Babies were divided into the following groups on the basis of the etiology of their thyroid gland failure:

(a) All TSH cases: all babies in Scotland who failed the neonatal screening program.
(b) DG: infants with ectopic, absent, or hypoplastic thyroids on imaging.
(c) DHG: infants with thyroid dysfunction and a thyroid gland *in situ* that was not hypoplastic but demonstrated abnormal isotope uptake.
(d) Unknown: cases with no clear etiology because infants did not undergo imaging or because they did not meet criteria for DG/DHG.

There were 11 cases of CHT where abnormal thyroid function was linked to maternal antibodies (blocking) or antithyroid drugs. These cases were excluded from analyses.

### The incidence of CHT according to season and year

Temporal trends were analyzed using the Poisson regression. Seasonal variation was assessed using a heterogeneity test.

### Space–time clustering methodology: rationale

Our premise was that DHG cases were more likely to contain patients with autosomal recessive disease than patients with DG. We anticipated a degree of overlap with, for example, some babies with DG having an underlying genetic defect typically attributed to DHG (15). However, a greater proportion of those in DG would have disease without an established etiology and would be more likely to demonstrate space–time clustering if environmental factors were to impact on thyroid gland development. Most babies in the unknown group were expected to have DG.

**FIG. 2.**
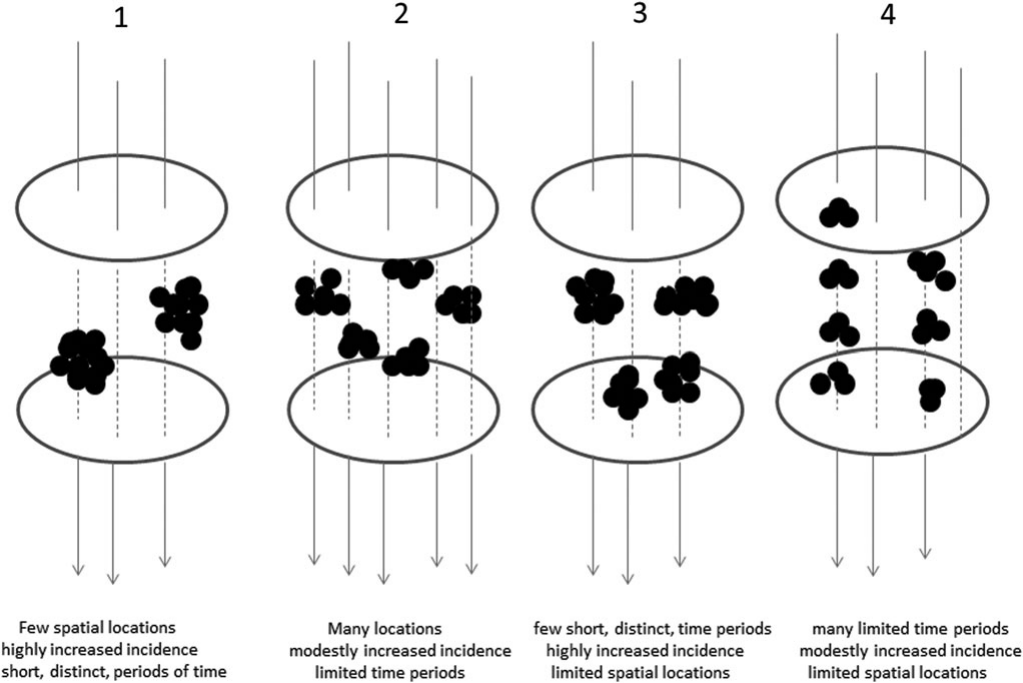
Illustration of the patterns of space–time clustering that can be observed.

Space–time clustering is an irregular distribution of specified disease cases simultaneously in space and time (Fig. 2). If a transient environmental agent, for example, an infection, is involved in etiology, then the distribution of case births may demonstrate space–time clustering. This would only result if the transient environmental agent occurred in mini-epidemics or if it only affected certain susceptible individuals. A ubiquitous environmental exposure would result in a homogeneous distribution of cases. Space–time clustering based on time and place of birth suggests that the etiologically relevant exposure occurred around the time of birth or *in utero* (16).

Many studies of space–time clustering have used the Knox test with fixed thresholds defining “closeness” in “space” and “time.” These limits are arbitrary. If the observed number of case pairs, which are close in space and time, is greater than the expected number, this suggests that case pairs close in space are also close in time (17,18). There are two methodological issues with the Knox test. Boundary problems may play a role as it may not be possible for certain cases to be close to other cases. Also, the thresholds are arbitrary, which resulted in multiple testing in other studies. A “nearest neighbor” (NN) is defined as the case that is situated most proximal to an index case. To adjust for effects of differing population birth densities, geographical distance can be replaced by the *N*th “nearest neighbor” (19). In this study, we used the “nearest neighbor” (NN) threshold method because this adjusts for population density variability.

### Space–time clustering analysis

A modification of a method based on *K*-functions was used (20). All analyses used a series of critical thresholds to define “closeness” in “time” and “space.” For “closeness in time,” the thresholds ranged from 0.1 to 1.5 years, in steps of 0.1 year. For “closeness in space,” the NN thresholds ranged from the distance to the 1st to the 15th nearest neighbor. Statistical significance was assessed by simulation, with the *p*-values obtained by randomly reallocated dates of birth to analysis cases. Analyses were conducted for all case pairs and also by sex and birth density level.

### Individual space–time clustering analysis

Kulldorff's scan statistic based on a space–time permutation model was used to identify individual clusters (21,22). The complete study region and time span were scanned by construction of a three-dimensional cylindrical moving window. The base of the cylinder represents two-dimensional geographical space and the height represents time. The base and height of this cylinder vary so that they include at most 50% of the entire time span and 50% of the entire geographical area. The variable base is centered on the postcode centroid of each case (23).

### Statistical significance

In all the analyses, a critical value of *p* < 0.05 was considered to indicate significance.

### Ethics

Informed consent for storage and later anonymized analysis and presentation of patients' data was prospectively collected since 2004. In cases born before 2004, parents, or patients aged 16 years and older, were approached to obtain retrospective consent for data storage and analysis.

## Results

There were 521 cases of CHT (Table 1). Serum TSH concentrations were available on 490 babies, with a median of 101 mU/L (minimum 7.2 mU/L, interquartile range [IQR] one 71 mU/L, IQR three 213 mU/L, maximum 1586 mU/L). More detailed analysis was not feasible because the upper limit of the TSH reference range differed between laboratories. Of the 521 babies, 55% had been characterized into DG/DHG. Of the babies with established DG, 116 (51%) had an ectopic gland, 116 (36%) had hypoplasia, and 30 (13%) had an absent gland, a distribution similar to values in the literature (24). As anticipated, two-thirds of DHG babies were female with equal numbers of males and females in the DHG group.

**Table 1.** Numbers of Babies by Subtype, Sex, and Birth Density

Category	No. of babies	Male/female	Higher/lower birth density
Dysgenesis	229	72/157	128/100^a^
Dyshormonogenesis	61	29/31^b^	35/26
Unknown	230	62/168	108/113^a^
Others	11	6/5	4/7
Total	531		

^a^ Location of birth not available for one case of dysgenesis and nine unknown cases.

^b^ Sex not available for one case.

### Change in incidence with time

**FIG. 3.**
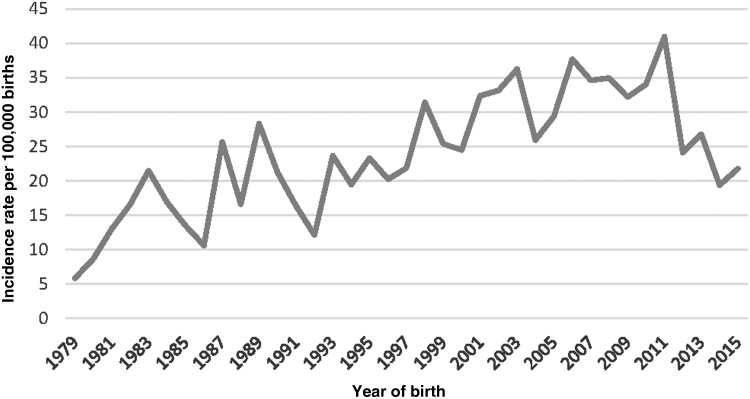
Plot of CHT incidence rates during the study time period. CHT, congenital hypothyroidism.

There was evidence of temporal increases for all cases, DG and DHG, but not for the unknown group (Table 2 and Fig. 3). There was no evidence of seasonal or sinusoidal variation. Although cutoffs were considered to be equivalent as assay methodology changed, there was a significant reduction in serum (as opposed to blood spot) TSH concentrations with time (*p* = 0.039). An upper limit in some local assays prevented analyses of disease severity.

**Table 2.** Analysis of Temporal Trends in Annual Congenital Hypothyroidism Incidence Rates (Increases or Decreases Expressed as per 10,000,000 Births), Together with 95% Confidence Intervals and *p*-Values

	Trend	*p*
All cases	24.9 [CI 16.9 to 33.0]	0.0001
Dysgenesis	32.0 [CI 9.7 to 44.3]	0.0001
Dyshormonogenesis	45.5 [CI 19.1 to 72.0]	0.0007
Unknown	−6.1 [CI −20.2 to 8.1]	0.3996

CI, 95% confidence interval.

### Clustering analyses

(a) All cases: there was no evidence of space–time clustering (*p* = 0.06, Table 3). Analyses by sex and birth density also did not show any space–time clustering.
(b) DG (*n* = 228): there was significant space–time clustering in DG (*p* = 0.007, Table 4). Analyses by birth density showed that clustering was restricted to areas with lesser birth density (*p* = 0.01). Space–time clustering was analyzed for DG subgroups, and there was clustering for hypoplasia (*p* = 0.02), but not ectopia (*p* = 0.24) nor agenesis (*p* = 0.35).
(c) DHG (*n* = 61): there was no evidence of space–time clustering in DHG (*p* = 0.35, Table 5).
(d) Unknown (*n* = 230): there was evidence of space–time clustering for all unknown cases (*p* < 0.001). There was significant clustering for pairs of cases involving females (*p* < 0.001), but not males (*p* = 0.06). Analyses by birth density showed significant clustering for areas with greater birth density (*p* < 0.001) and also from areas with lesser birth density (*p* = 0.01).

### Individual cluster analyses

The space–time clustering analysis conducted among all cases of DG found that there was one large significant cluster occurring during the period from January 1, 1983, to October 31, 1991 (observed cases [O] = 20, expected cases [E] = 5.75, O/E = 3.48, *p* = 0.005). For unknown cases, there were two large significant clusters occurring during the period from March 1, 1983, to October 31, 1990 (O = 3, E = 12.54, O/E = 2.63, *p* = 0.001) and from April 1, 2010, to August 31, 2013 (O = 12, E = 2.00, O/E = 6.01, *p* = 0.003).

**Table 3.** Results of the *K*-Function Analysis for Space–Time Clustering for All Cases by Time of Birth (*p*-Value)

Group	*p*
All case pairs	0.06
“Male: any” case pairs	0.11
“Female: any” case pairs	0.09
“Greater birth density: any” case pairs	0.09
“Lesser birth density: any” case pairs	0.10

*p*-Value obtained by simulation (999 runs) with dates of birth randomly reallocated to the cases in the analysis. Cases are close in time if dates of birth differ by <*t*, where *t* is in the range of 1–15 months. Cases are close in space if either is within the distance to the *N*th nearest neighbor of the other, where *n* is in the range of 1–15 (birth locations).

**Table 4.** Results of the *K*-Function Analysis for Space–Time Clustering for Cases of Dysgenesis by Time of Birth (*p*-Value)

Group	*p*
All case pairs	0.007
“Male: any” case pairs	0.04
“Female: any” case pairs	0.003
“Greater birth density: any” case pairs	0.05
“Lesser birth density: any” case pairs	0.01

*p*-Value obtained by simulation (999 runs) with dates of birth randomly reallocated to the cases in the analysis. Cases are close in time if dates of birth differ by <*t*, where *t* is in the range of 1–15 months. Cases are close in space if either is within the distance to the *N*th nearest neighbor of the other, where *n* is in the range of 1–15 (birth locations).

**Table 5.** Results of the *K*-Function Analysis for Space–Time Clustering for Cases of Dyshormonogenesis by Time of Birth (*p*-Value)

Group	*p*
All case pairs	0.35
“Male: any” case pairs	0.86
“Female: any” case pairs	0.50
“Greater birth density: any” case pairs	0.76
“Lesser birth density: any” case pairs	0.52

Cases are close in time if dates of birth differ by <*t*, where *t* is in the range of 1–15 months. *p*-Value obtained by simulation (999 runs) with dates of birth randomly reallocated to the cases in the analysis. Cases are close in space if either is within the distance to the *N*th nearest neighbor of the other, where *n* is in the range of 1–15 (birth locations).

## Discussion

We have identified space–time clustering in babies with CHT due to thyroid DG but not DHG in Scotland. Space–time clustering is transient clustering, which does not reflect genetic variation and is different to seasonality or temporal changes in incidence. Space–time clustering suggests that transient environmental factors may be involved in DG etiology. One would not expect the same pattern of clustering in babies with DHG except in the context of twins or siblings born at times that are very close together, which was not observed.

The absence of space–time clustering in DHG babies fits with our hypothesis. There was evidence of space–time clustering in the unknown group of cases, and the lack of clustering of cases of DHG may have diluted the finding for the entire cohort. A similar dilution has been noted in a large national study of space–time clustering of childhood cancer where there was clustering among individual groups, but not overall (25). The fact that space–time clustering occurred in the context of females in the “unknown” group may reflect a preponderance of females with DG as one might predict. The analyses of individual clusters showed that there was a mixture of different scenarios (Fig. 2), although only one reached formal statistical significance for DG cases, while two reached significance among unknown cases. There were differences in the nature of space–time clustering in Scottish patients when compared with our earlier study (8), but the population distribution and nature of environmental factors will be different.

Seasonal variation in CHT has been identified by some authors (26) but not by us or others (27). The absence of a clear seasonal pattern in CHT incidence coupled with differences in CHT incidence between ethnic groups suggests that environmental factors might not be closely involved in CHT etiology (27,28), although analyses did not extend to space–time clustering (26).

We noted an increase in CHT incidence by year, and this could be linked to factors such as case ascertainment or change in screening methodology. A rising incidence of CHT has been described in many nations and has frequently been attributed to a fall in TSH screening threshold or a change in assay methodology. We did see evidence to suggest that methodology change was associated with a change in the number of CHT “cases” detected, but this does not preclude a true alteration in incidence of babies with abnormal thyroid gland function (3,4). A change in threshold might preferentially detect cases of DHG, although both DHG and DG can be associated with a mild biochemical phenotype (6).

Environmental factors such as iodine can affect thyroid gland dysfunction incidence. Recent data have suggested that parts of the United Kingdom appear to be iodine sufficient, although one would expect a suboptimal iodine status to impact on the number of babies identified as having mild CHT with a gland *in situ* and increased isotope uptake and hence to increase the number of “DHG” cases rather than DG (29).

A key consideration when interpreting our study findings is whether they are biologically plausible. Halogenated organochlorines and pesticides can disrupt thyroid function, and polychlorinated biphenyls and their metabolites and polybrominated diethyl ethers bind to thyroid transport proteins and are associated with placental thyroid hormone concentrations (30–32). Free thyroxine concentrations and thyroid gland volume were linked to exposure to a fungicide (mancozeb) with the magnitude of the relationship affected by iodine status (33). Bisphenol, widely used in industry, is common in the natural environment and can impact on the transcription of genes involved with thyroid gland development (34). A Japanese study found a correlation between organochlorine insecticides and dioxin-like chemicals in the milk of mothers who had given birth to infants with CHT (35). The occurrence of such chemical exposures is likely to be dependent on atmospheric conditions and would be expected to exhibit a transient occurrence in space and time. Dioxin exposure was reported to be associated with higher neonatal TSH values in pregnancies many years after the Seveso accident in Italy (36). The thyroid gland develops at an early stage *in utero*, and space–time clustering in the gland hypoplasia subgroup of DG is compatible with an environmental disruptor at a relatively early stage of pregnancy.

Space–time clustering suggests the involvement of a spatially varying and transient environmental agent or agents in etiology. Such agents could include pollutants, or pesticides, which would be expected to occur in more rural communities. Our analyses found that significant evidence of space–time clustering of DG was limited to areas with lower birth density, but environmental agents will not exclusively be focused here, and so, the presence of clustering linked to higher as well as lower birth density in the unknown group is plausible. Although the Scottish fertility rate has decreased from 1.84 per 1000 female population in 1979 to 1.56 per 1000 female population in 2015 (www.nrscotland.gov.uk), the statistical methodology has adjusted for birth density.

It is important to highlight the fact that genetic factors may be more important in DG etiology than previously thought. In one Italian study, a next-generation sequencing methodology identified oligogenic involvement of candidate genes in 23% of patients with a thyroid gland *in situ* or DG (37). The etiology of most cases of DG was still unclear.

There are study limitations. We were able to look at all cases of CHT, but only 55% could be definitively characterized, and we did not undertake comprehensive genetic analyses. Hence, there may be areas in parts of Scotland where clustering of babies with CHT due to DG was not identified. Differences in the level of patient characterization may explain some of the discrepancies noted between analyses in the DG/DHG and the “unknown” category. However, this limitation would reduce the likelihood of clustering being identified and hence increase the likelihood of a type II statistical error. We considered whether changes in imaging practice could account for case clustering, but there has not been any appreciable change during the study period. Changes in assay methodology could alter the number of cases above a certain threshold, despite attempts to align old and new techniques but would not result in space–time clustering.

There are also methodological issues to consider with the current study. We were not able to take into account changes in the birth rates over the years, changes in population density, population mobility, and potential ethnic background changes due to immigration. However, changes in birth rates would not lead to space–time clustering. Variations in population density have been accounted for and longer-term changes in population density would not lead to space–time clustering. It is possible that population mobility may lead to space–time clustering, but this seems unlikely as the results were not the same for different diagnostic groups. The test is biased if there are population shifts during the time period, such as when the population grows or declines at different rates in different parts of the study period (38). Our analyses provide a description of apparent space–time clustering, but we believe that variations in population growth are unimportant in the present data set.

In summary, environmental factors may be involved in the etiology of CHT due to DG. The fact that significant clustering occurred in DG associated with gland hypoplasia is biologically plausible if these factors impair thyroid gland development. The incidence of CHT varies globally, and while this may be linked to complex genetic factors, an environmental exposure or exposures may also be important.
